# Machine Learning Methods as a Cost-Effective Alternative to Physics-Based Binding Free Energy Calculations

**DOI:** 10.3390/molecules29040830

**Published:** 2024-02-13

**Authors:** Nupur Bansal, Ye Wang, Simone Sciabola

**Affiliations:** Biotherapeutic and Medicinal Sciences, Biogen, 225 Binney Street, Cambridge, MA 02142, USA; ye.wang@biogen.com (Y.W.); simone.sciabola@biogen.com (S.S.)

**Keywords:** benchmarking, free energy, ligands, receptors, screening assays

## Abstract

The rank ordering of ligands remains one of the most attractive challenges in drug discovery. While physics-based in silico binding affinity methods dominate the field, they still have problems, which largely revolve around forcefield accuracy and sampling. Recent advances in machine learning have gained traction for protein–ligand binding affinity predictions in early drug discovery programs. In this article, we perform retrospective binding free energy evaluations for 172 compounds from our internal collection spread over four different protein targets and five congeneric ligand series. We compared multiple state-of-the-art free energy methods ranging from physics-based methods with different levels of complexity and conformational sampling to state-of-the-art machine-learning-based methods that were available to us. Overall, we found that physics-based methods behaved particularly well when the ligand perturbations were made in the solvation region, and they did not perform as well when accounting for large conformational changes in protein active sites. On the other end, machine-learning-based methods offer a good cost-effective alternative for binding free energy calculations, but the accuracy of their predictions is highly dependent on the experimental data available for training the model.

## 1. Introduction

In silico protein–ligand binding affinity calculations have become a powerful tool in the computational chemist’s toolbox. If predicted correctly, in silico estimates of binding affinity can significantly accelerate drug discovery projects in the pharmaceutical industry by focusing experimental efforts on tight-binding leads [1,2,3,4]. Physics-based methods have dominated the field since the beginning and have proven to be highly successful, but they pose problems with forcefield choice, sensitivity to ligand parametrization, and adequate sampling [5]. Many academics and industrial groups have implemented their own tweaks to conventional physics-based methods with the intent of improving protein–ligand binding free energy calculations [6,7,8,9,10]. While the methods are numerous, they can be largely characterized into three different categories, with the simplest being end-point methods, such as docking, which involves the calculation of energies of static structures [11,12,13,14]. These methods involve exploring the possible orientations and conformations of ligands in mostly static binding sites and finding the most favorable binding pose based on an empirical scoring function. The performance of these methods relies hugely on the forcefield underneath, as there is no sampling or minimization involved. Another class of methods that includes the Molecular Mechanics Poisson–Boltzmann/Surface Area (MM-PBSA) and Molecular Mechanics–Generalized Born/Surface Area (MM-GBSA) methods involve limited sampling and evaluation of the absolute binding free energies of a protein and ligand before and after binding. These methods are slightly more complex than docking calculations and offer a higher level of thermodynamic detail of the system by considering solvation/desolvation along with electrostatics and van der Waals interactions. To account for the effects of solvation, they used an implicit solvent model (Generalized Born in the case of GB and Poisson–Boltzman for PB) and considered the changes in the solvent’s accessible surface area upon binding to account for the contributions of desolvation [15,16,17,18]. The binding free energy was calculated as the difference between the free energy of the ligand–receptor complex and the sum of unbound ligand and receptor free energies. While they were successfully used to predict binding modes and conduct structural stability determination, virtual screening, and ligand optimization, one of the major limitations of such methods is the approximation introduced by the implicit solvent model. The following reviews discussed the MM-PBSA and GBSA methods in more detail [19,20,21]. The most complex forms of methods are alchemical binding free energy calculations that involve enhanced sampling, and they are theoretically the most accurate out of the three different categories of methods [1,3,22,23,24,25,26,27,28,29,30]. Alchemical methods are pathway-based methods that smoothly transform the state of a system from one to another over a series of intermediate alchemical states, estimating the change in free energy associated with the transformation. They are simulation-based methods and are particularly valuable in studying the binding of ligands to proteins, conformational changes associated with protein binding, and chemical reactions. There are multiple variations of alchemical methods, of which free energy perturbation (FEP) and thermodynamic integration (TI) are most commonly employed in understanding protein–ligand interactions. Although alchemical free energy simulation methods include the most rigorous physics-based workflows for calculating the difference in free energy between a ligand and a protein, setting up, running, and analyzing these simulation-based methods poses significant challenges because of the complex nature of the computational algorithms involved, requiring extreme care to ensure the accuracy and reliability of the results. Additionally, they are computationally resource-intensive, and the accuracy of the predictions relies extensively on the quality of the forcefield parameters, sampling time, and method employed. It is also crucial to make sure that simulations have converged overall, making the process extremely cumbersome and impractical for application in the fast-paced environment of pharmaceutical drug discovery. To improve throughput, highly automated GPU-enabled free energy workflows such as FEP+ [2,31] and Thermodynamic Integration (TI) [32] have been established. The inclusion of enhanced sampling algorithms and easy-to-use user-interface-based workflows has increased the rigor and throughput of simulation-based methods and made them convenient for application in drug discovery pipelines [5,7,10,33,34,35,36]. However, the required computational cost still represents the most limiting factor in the daily usage of these methods. Recent advances in the field of machine learning, particularly those related to deep learning methods, have increased interest in their application for protein–ligand binding affinity predictions [37,38,39,40,41,42,43,44,45,46]. Machine-learning-based methods offer advantages over classical physics-based methods in terms of their flexibility to adapt to different types of data by using a more generalized molecular representation that does not impose an explicit functional form like physics-based methods do [47], making them a powerful alternative when dealing with complex nonlinear molecular interactions. As these methods are built on large datasets of experimentally determined binding affinities provided in the training sets, they are able to capture patterns in data that might be more challenging for physics-based methods to model explicitly. At the same time, their performance relies heavily on the features and model of choice, not to mention the quality of the data fed to build the model. Nevertheless, their efficiency allows for quicker and more scalable free energy predictions when compared to their physics-based counterparts.

In this study, we retrospectively evaluated several commercial physics-based methods and a machine learning method using a dataset containing 172 ligands from four different active internal therapeutic projects spread across five different congeneric series with the goal of observing the trends and challenges faced by leading methodologies on real-world data. We were able to show the strengths and limitations of the different free energy methods, providing a better understanding of which tool might perform best at specific stages of the drug discovery process, ultimately impacting the efficiency and speed of the molecular optimization cycle. It is important to acknowledge that our retrospective evaluation is by no means exhaustive but based on a subset of free energy methods that were available to us. Despite this constraint, we believe that our findings are generalizable to the extended set of methods described in the broader literature.

## 2. Results and Discussion

**Figure 1:**
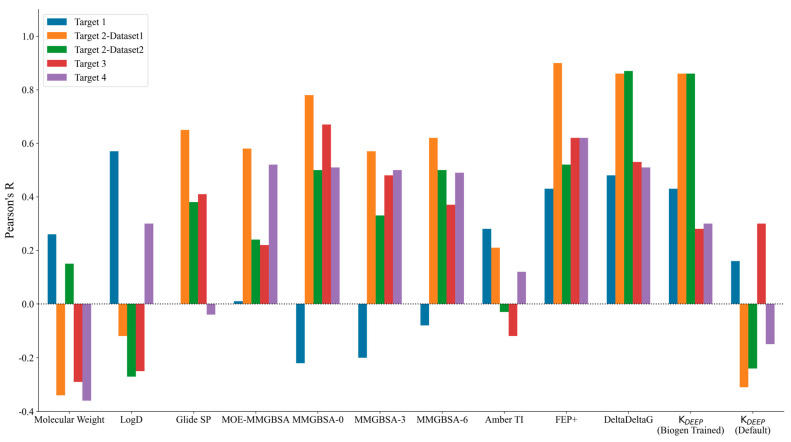
Pearson’s R values obtained by correlating experimental binding affinity with the predicted potencies for each method used (listed on the *x*-axis). A larger positive R value (depicted as a higher bar) indicates a better correlation with the experimental data. The colors used for the different targets are shown in the legend above the plot.

The performance of the different methods used was measured in terms of Pearson’s R correlation coefficient between the experimental binding affinity and the calculated binding free energy values. Figure 1 highlights the graphical comparison of Pearson’s R values observed for each target across the different methods (shown on the *x*-axis) used in this study. A higher positive bar indicates a better correlation, while a negative bar indicates anticorrelation. Table S1 in the Supporting Information lists the Pearson’s R values observed for each target.

The physicochemical properties, such as the molecular weight and clogD, did not show any significant correlations for any of the targets except for Target 1. Interestingly, the best correlation for Target 1 was observed with respect to clogD with Pearson’s R of 0.57. Among the physics-based methods, only TI and FEP+ showed a limited correlation for Target 1, with Pearson’s R values of 0.28 and 0.43, respectively. The trend was somewhat justified for Target 1, as it was an enzyme with a fairly large hydrophobic pocket and with three major observed binding modes for different ligand project series, and it was primarily driven by non-specific interactions. Although we started from a high-resolution co-crystal structure and picked close analogs within the same chemical series for this study, it is possible that a larger amount of sampling is required to obtain accurate poses and binding affinity predictions, as evidenced by the improved binding affinity correlations from the FEP+ and TI methods in comparison with those of the other physics-based methods with little to no receptor sampling. Targets 2–4 belonged to the kinase family and showed decent correlations for the physics-based methods. The Glide SP docking score did quite well for Target 2–Dataset1 with Pearson’s R of 0.65, but it showed very little to no signal for the remaining targets. Prime MM-GBSA with no protein flexibility consistently showed the best correlations among all of the MM-GBSA methods employed in the study. Counterintuitively, adding protein flexibility in the MM-GBSA calculations did not show improvement in Pearson’s R correlation for any of the targets, as could be observed by comparing Prime’s MM-GBSA correlations when allowing protein sampling within 0, 3, and 6 Å from any ligand atoms. The implementation of MM-GBSA in MOE performed worse than Prime for Target 2 (both datasets) and Target3, and it showed comparable results to those for Target 4.

Among the simulation-based free energy methods, we benchmarked the performance of FEP+ and the MOE implementation of Amber-TI. In our study, FEP+ clearly outperformed every other physics-based method, while the results of Amber-TI in MOE were surprisingly inconsistent. A possible explanation could be the Amber–EHT forcefield combination or the newly optimized alpha and beta parameters implemented in MOE for the soft-core potential. As we did not run Amber-TI calculations on these systems directly using the Amber package, we are unsure of the possible source of error. Overall, comparing the results of Prime MM-GBSA with no protein sampling and FEP+, we can state that for kinase Targets 2–4, Prime performed equally well and offered a good trade-off in terms of computational time and resources compared to FEP+. The major difference was observed for Target 1, for which the enhanced sampling introduced by FEP+ yielded a significant enhancement in binding affinity correlation.

**Figure 2:**
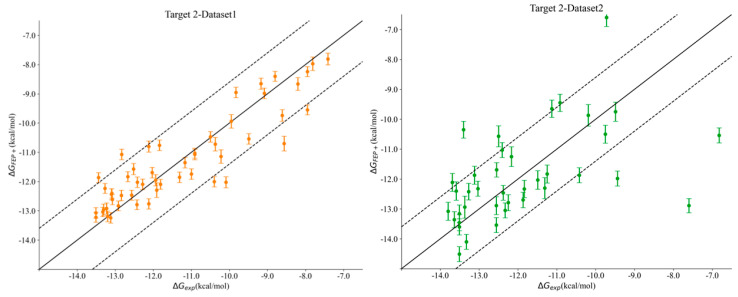
Correlation plots obtained for Target 2–Dataset1 (**left**) and Target 2–Dataset2 (**right**) using FEP+. The dashed line is at 1.4 kcal/mol (1 log unit difference).

Interestingly, we noticed a stark difference in Pearson’s R correlation between Target 2–Dataset1 and Target2–Dataset2. As we previously mentioned, both datasets shared the same kinase active site and the same hinge–binder scaffold. The only difference between the two sets resided in the medicinal chemistry optimization strategy, where the ligands within Dataset1 were optimized towards the solvent-exposed region, while the ligands in Dataset2 were optimized for their interactions towards the P-loop. In general, the physics-based methods from the end state and the simulation-based methods consistently predicted Dataset1 with much better accuracy than that obtained for Dataset2, with FEP+ obtaining an R value of 0.90 for Dataset1 and 0.50 for Dataset2. Figure 2 highlights the correlation plots obtained for Target 2–Dataset1 and Target2–Dataset2 with FEP+. The dashed line in the plot was drawn at 1.4 kcal/mol (roughly 1 log unit), and it can be clearly seen that 90% of the compounds for Dataset1 were within an error range of 1.4 kcal/mol, and those that were outside the dashed line were still predicted to be quite close to the experimental binding affinities. This difference was also reflected in the pairwise RMSE values obtained with FEP+ (listed in Table S2 in the Supporting Information), which were 1.16 kcal/mol for Dataset1 and 2.6 kcal/mol for Dataset2. The correlation plots obtained with FEP+ for all of the remaining targets are provided in Figure S2 in the Supporting Information. Targets 3 and 4 had changes under the P-loop region as well, and their prediction accuracy was similar to that for Target 2–Dataset2 with all of the physics-based methods, thus supporting the idea that the conformational sampling of large loop motions is still challenging.

**Figure 3:**
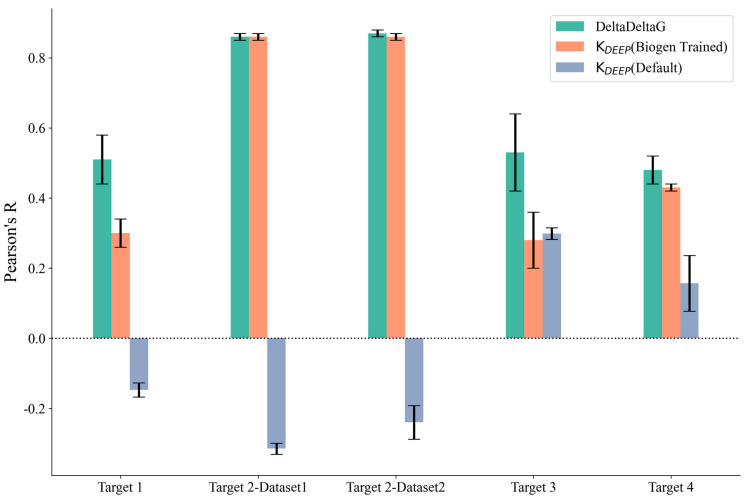
Pearson’s R and standard deviation (shown as error bars) for the machine learning methods used. K_DEEP_ (BIOGEN Trained) (orange), K_DEEP_ (Default) (gray), and DeltaDeltaG (green).

We further explored the potential for machine-learning-based methods to predict the binding affinity for our set of 172 ligands across the four protein targets. We applied two versions of K_DEEP_ using the standard model, which did not require retraining on our internal dataset (referred to as K_DEEP_ (Default)), and by retraining the default model on our internal data (referred to as K_DEEP_ (Biogen Trained)). As can be observed in Table S1 and Figure 3, the Pearson’s R value for the K_DEEP_(Default) method was worse than that for than K_DEEP_ (Biogen Trained), which was expected because the retrained model could directly leverage project-based assay information that was not available for the default model, which was only trained on publicly available binding affinity determinations. In addition to K_DEEP_, we also explored the DeltaDeltaG method (retrained on our internal project assay data), which was specifically designed to predict relative binding free energies for congeneric series. To understand the variability in the predictions, both K_DEEP_ and DeltaDeltaG were assessed using five independent runs, and the average Pearson’s R values across the five runs are reported in Table S3. The results were quite consistent across all of the runs, and the standard deviation obtained was less than 0.1 for all of the methods (Figure 3). Pearson’s R values for DeltaDeltaG and K_DEEP_ on our internal trained sets were comparable, with DeltaDeltaG performing slightly better. This could be explained by the validation task being more aligned with the model-building strategy implemented in DeltaDeltaG, where pairs of protein–ligand voxelizations and their ddG values were used to retrain the two-legged neural network, while K_DEEP_ was more general, was designed for absolute binding affinity prediction, and was not restricted to congeneric series of ligands. Overall, Pearson’s R value for DeltaDeltaG for all of the targets was comparable to the scores of FEP+ and prime MM-GBSA (rigid protein). The specific details of each run of DeltaDeltaG for all targets, including the prediction time, number of compounds used in the training set, and prediction accuracy, are provided in Table S3 in the Supporting Information. Given that DeltaDeltaG achieved comparable accuracy to that of the best-performing physics-based methods while generating average protein–ligand binding affinity predictions in under a minute, as opposed to the cumbersome and expensive setup required by the latter, it became evident that, for projects with large high-resolution experimental datasets, utilizing machine learning algorithms represents a significantly more cost-effective alternative.

## 3. Methods

### 3.1. Datasets

We selected 172 ligands from internal projects targeting 4 different protein targets. Target 1 was an enzyme with a relatively large and hydrophobic binding pocket adjacent to a bimetallic nucleophilic catalytic site, while Targets 2–4 were structurally diverse protein kinases where binding specificity was dictated by a complex interplay of hydrophobic, hydrogen bonding, and electrostatic interactions between amino acid residues on the kinase orthosteric site and the substrate. Although we experimentally observed multiple binding modes for compounds from different series for Target 1, in this study, we selected ligand analogs from the most explored chemical series that resulted in the highest-resolution co-crystal structure. For one of the kinase projects (Target 2), we selected compounds according to different optimization strategies. Target 2–Dataset1 included a set of chemical analogs in which the hinge binder scaffold and the P-loop interacting decoration were shared across all of the ligands, while the changes were directed towards the solvent-exposed region. Target 2–Dataset1 included a set of chemical analogs in which the hinge binder scaffold and the solvent-exposed R-group were constant across all of the ligands, and the changes were made under the P-loop region. The choice of compiling different benchmark sets for Target 2 was aimed at decoupling the limitations of physics-based simulation methods regarding the accurate sampling of the dynamics of the P-loop region from the forcefield accuracy.

**Figure 4:**
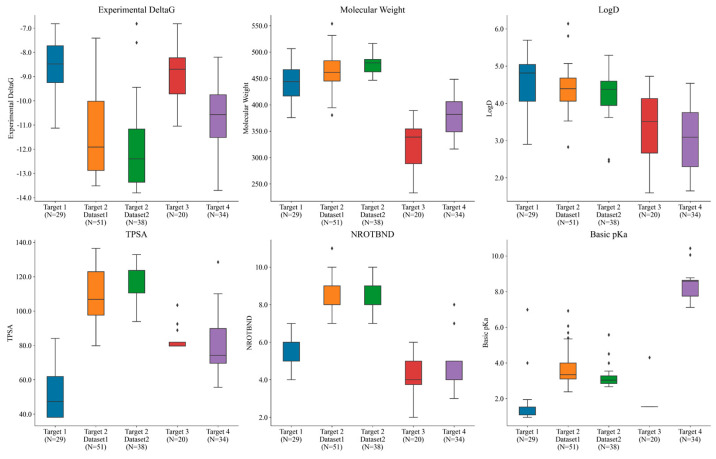
Different targets used in the study spanning across different ranges of experimental potencies, molecular weights, logD (lipophilicity) values, TPSA (total polar surface area), numbers of rotatable bonds, and basic pKa values. N represents the number of compounds in each set. Black dots outside the box plot show the outliers observed in each case.

Overall, the compounds in each set were manually selected with an emphasis on exploring activity cliffs [48] (i.e., pairs of structurally similar compounds with large differences in potencies measured against the same target). Figure 4 shows the range of experimental potencies observed across the five selected ligand series, as well as some basic physicochemical property profiles. The number (N) of compounds in each dataset is also reported on the *x*-axis. The range of experimental affinities was quite wide (as can be seen in Figure 4), spanning from 3.5 to 5 log units across all of the targets. Figure 4 also shows how the selected compounds were picked to sample a diverse space of physicochemical properties. The average molecular weight across the five datasets ranged from 320 to 480 Daltons, while the lipophilicity (logD) ranged between 2 and 5.5. Additionally, the total polar surface area (TPSA) ranged from 38 to 136, the number of rotatable bonds ranged from 2 to 11, and the basic pKa ranged from 1 to 10.5 across all targets. Overall, the five datasets consistently positioned the compounds within a favorable CNS space.

### 3.2. Computational Details

**Protein and ligand preparation**. The starting protein–ligand structures were taken from in-house co-crystal structures with a high resolution (<2.5 A). The proteins were prepared using the Protein Preparation Wizard accessible from the Maestro interface (Schrodinger 2019-3 suite) at a pH of 7.0 [49]. The hydrogen atom positions were optimized using the OPLS3e forcefield [50,51,52]. The optimization was performed in the absence of the ligand. Crystal waters that did not interact with the ligand were removed from the receptor structures. Only hydrogen atoms were minimized, while the heavy atoms were kept fixed. Ligands were prepared using the LigPrep utility in Schrodinger with the OPLS3e forcefield. Epik was used to determine the protonation and the most favorable tautomer state of ligands [53,54]. Stereochemistry was retained from the input ligand structures.

**Computational methods**. We used a range of different commercially available physics-based methods, including Glide SP [11] for docking, MM-GBSA from prime [55] and MOE [32], alchemical free energy simulations using FEP+ [2,31,56], and Thermodynamic Integration (TI) from MOE. Along with these, two machine learning methods, namely, K_DEEP_ and DeltaDeltaG, were evaluated. As the physics-based methods that we used were not stochastic in nature like machine learning methods, they were run just once, while we performed 5 runs each for K_DEEP_ and DeltaDeltaG. The following sections discuss the methods in further detail.

**Docking**. Protein grids were generated using the *Receptor Grid Generation* utility in Glide to assign the ligand position in the binding pocket. The docking of ligands was performed using the standard precision (SP) ligand docking module in Glide after the preparation step [11]. Core constraints were applied during docking to restrain the core scaffold on ligands. For a fair comparison, we used glide-docked poses as the starting bound conformation for all other methods.

**MMGBSA**. The Molecular Mechanics–Generalized Born/Surface Area (MM-GBSA) method is slightly more complex than docking calculations, as it computes ligand strain and solvation/desolvation and, therefore, adds more accuracy to the protein–ligand binding prediction. Prime MMGBSA calculations were performed directly in the Maestro interface using three different settings for protein sampling: rigid protein (0 Å sampling), 3 Å protein sampling, and 6 Å protein sampling. The solvation model used was VSGB 2.1 with the OPLS4 forcefield [52]. Hierarchical sampling was carried out to perform systematic sampling of ligand positions, orientations, and conformations, along with receptor residues. MM-GBSA calculations were also performed in the MOE interface using default settings [32].

**Free Energy Perturbation (FEP+)**. FEP+ calculations were set up using Schrodinger2017-4 [2,6,31]. The FEP mapper module implemented in Desmond was used to set up the calculations using the SPC water model. The torsion parameters for the ligands that were not included in the OPLS3e forcefields were generated using a forcefield builder first. FEP+ jobs were submitted using the default settings with a grand canonical ensemble with 0.02 ns of equilibration time and 5 ns of simulation time. Twelve lambda windows were used for default perturbations, and 24 were used for charged perturbations.

**Thermodynamic Integration (TI)**. Thermodynamic Integration was run using a plugin available in the MOE interface with the Amber10:EHT forcefields [32]. A default temperature of 300 K, a pressure of 101 kPa, and a sodium chloride salt concentration of 0.1 M were used. Equilibration times of 3 ns per transformation edge and 0.4 ns per window were used. The minimum sampling time in each window was set to 5 ns and 30 windows were used, collecting a total simulation time per ligand of 150 ns. Further details of the TI jobs are provided in Figure S1, which shows a snapshot of the TI interface with the settings used to run the calculations.

**DeltaDeltaG and K_DEEP_**. Internally, we had access to two 3D convolutional neural network methods for calculating binding free energies. K_DEEP_ was designed to predict absolute binding free energies [57], and DeltaDeltaG [58] was specifically designed to tackle relative binding free energies for congeneric series of ligands. Both the K_DEEP_ and DeltaDeltaG models were trained on PDBbind [59] and BindingDB [38], and they were made accessible through Playmolecule.org. The original models were augmented using independent project data consisting of similar analogs across the 4 targets that were not part of the validation sets. K_DEEP_ was run using two settings: the standard model without internal re-training (referred to as K_DEEP_ (Default)) and the model retrained on our internal data (referred to as K_DEEP_ (Biogen Trained)). DeltaDeltaG was run directly using the internally retrained models. To check for uncertainty in prediction, we performed 5 runs each for K_DEEP_ and DeltaDeltaG. Table 1 lists the numbers of compounds used to retrain the original models provided via the Playmolecule application. It also shows the maximum similarity (Tanimoto) average and standard deviation between each compound in the test set and all of the compounds in the training set.

## 4. Conclusions

The ability to rank and score different potential drug candidates against a protein target remains one of the most elusive challenges in drug discovery, which is primarily due to the intricate nature of biological systems. Accurate in silico binding free energy methods can improve our ability to navigate the complexities of the drug discovery process, as they offer a faster and cost-effective way to screen large compound databases and narrow down the pool of potential drug candidates for experimental validation. Physics-based in silico free energy prediction methods have dominated the field for the longest time and have recently shown tremendous improvement with the advent of FEP+, but they bring their own challenges, including but not limited to system preparation, simulation length, forcefield parameterization, computational cost, and so on. Machine learning methods provide a valuable alternative in advanced stages of drug discovery projects, particularly when large experimental datasets are available. In this study, we evaluated state-of-the-art binding free energy methods that were accessible to us against five datasets that spanned across four internal therapeutic targets. Overall, the performance of the physics-based methods across all protein targets could be largely categorized as Glide SP docking showing a lower correlation than that of Prime MM-GBSA (with the rigid protein being slightly better) and FEP+ affording the best performance. Considering the computational cost, using Prime MM-GBSA with limited protein flexibility offers a good trade-off compared to FEP+. FEP+ gave good correlations when increased sampling was needed (e.g., Target 1) compared to MM-GBSA, but it did not do as well when accounting for P-loop flexibility (Target 2–Dataset2, Target 3, and Target 4). We evaluated two machine learning algorithms, K_DEEP_ and DeltaDeltaG, alongside the physics-based methods. We compared the two versions of K_DEEP_—the standard model without retraining on our internal dataset versus the augmented model obtained by retraining the default model on internal data. As expected, the prediction accuracy of the standard model trained on publicly available binding affinity determinations was substantially worse than that of the retrained model, which could leverage direct project-based assay information. After training both of these supervised methods (K_DEEP_ and DeltaDeltaG) with our internal project data, we observed comparable performance to that of FEP+, especially with DeltaDeltaG, suggesting that machine learning methods can be used as a cost-effective alternative to physics-based simulation methods, though their application and performance depend on the availability of relatively large amounts of curated experimental binding affinity data.

## Figures and Tables

**Table 1:** Composition of training and test sets across all five targets. The average and the standard deviation of the maximum similarity of each compound in the test set to all the compounds in the training set are also shown.

	Train	Test	Max Similarity (µ)	Max Similarity (σ)
Target 1	52	29	0.76	0.06
Target 2–Dataset1	72	51	0.77	0.07
Target 2–Dataset 2	158	38	0.84	0.07
Target 3	57	20	0.83	0.11
Target 4	195	34	0.81	0.09

## Data Availability

The data to reproduce plots is accessible through the authors, however the structures of proteins, compounds and docked poses are confidential and cannot be disclosed.

## Supplementary Materials

The following supporting information can be downloaded at: https://www.mdpi.com/article/10.3390/molecules29040830/s1, Figure S1: Amber-TI conditions used for all the targets in MOE interface; Figure S2: Correlation plots obtained from FEP+ for Targets 1, 3 and 4; Table S1: Lists the mean Pearson’s R value obtained for each Target for all the methods that were used in this study; Table S2: Lists the Pearson’s R^2^ and pairwise RMSE values obtained via FEP+ for each Target; Table S3: Details of each run of DeltaDeltaG for each Target.
