# Supplementary material for: Machine Learning Methods as a Cost-Effective Alternative to Physics-Based Binding Free Energy Calculations
Source: Molecules. 2024 Feb 13;29(4):830. doi: 10.3390/molecules29040830 (PMC10893267; doi:10.3390/molecules29040830)
Supplement: Supplementary file 1 [file molecules-29-00830-s001.zip › molecules-2810464-supplementary.pdf]

Supporting Information

Machine Learning Methods as a Cost-Effective Alternative to Physics-Based Binding Free Energy Calculations

Nupur Bansal *, Ye Wang and Simone Sciabola

Biotherapeutic and Medicinal Sciences, Biogen, 225 Binney Street, Cambridge, MA 02142, USA
* Correspondence: nupur.bansal@biogen.com; Tel.: +1-(617)-914-0719

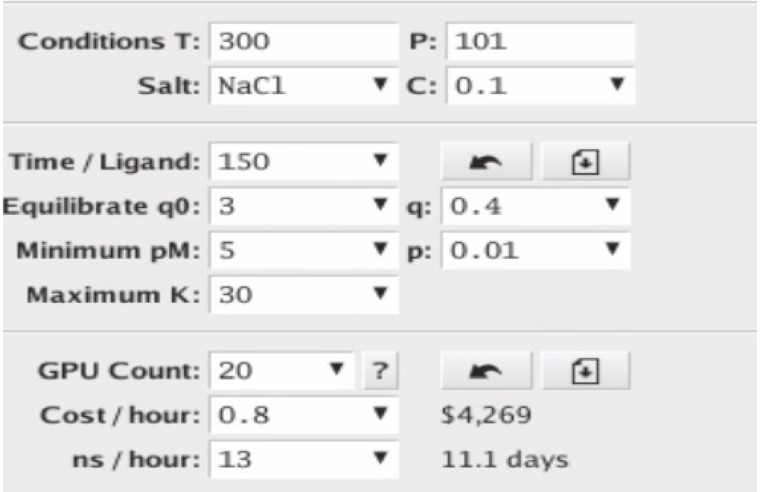

Figure S1. Amber-TI conditions used for all the Targets in MOE interface.

Table S1. Lists the mean Pearson's R value obtained for each Target for all the methods that were used in this study.

	Target 1 (N=29)	Target 2-Dataset 1 (N=51)	Target 2-Dataset 2 (N=38)	Target 3 (N=20)	Target 4 (N=34)
Mol wt.	0.26	-0.34	0.15	-0.29	-0.36
cLogD	0.57	-0.12	-0.27	-0.25	0.30
Glide SP	0.00	0.65	0.38	0.41	-0.04
MOE MMGBSA	0.01	0.58	0.24	0.22	0.52
MMGBSA-0	-0.22	0.78	0.50	0.67	0.51
MMGBSA-3	-0.20	0.57	0.33	0.48	0.50
MMGBSA-6	-0.08	0.62	0.50	0.37	0.49
Amber TI	0.28	0.21	-0.03	-0.12	0.12
FEP+	0.43	0.90	0.52	0.62	0.62
DeltaDeltaG	0.48	0.86	0.87	0.53	0.51
KDEEP (Biogen Trained)	0.43	0.86	0.86	0.28	0.30
KDEEP (Default)	0.16	-0.31	-0.24	0.30	-0.15

Table S2. Lists the R^2 and pairwise RMSE from FEP+ observed for each Target.

	N	FEP+ R^2	FEP+ RMSE (pairwise)
Target 1	29	0.72	1.47
Target 2-Dataset1	51	0.87	1.16
Target 2- Dataset 2	38	38	2.46
Target 3	20	20	1.80
Target 4	34	34	1.66

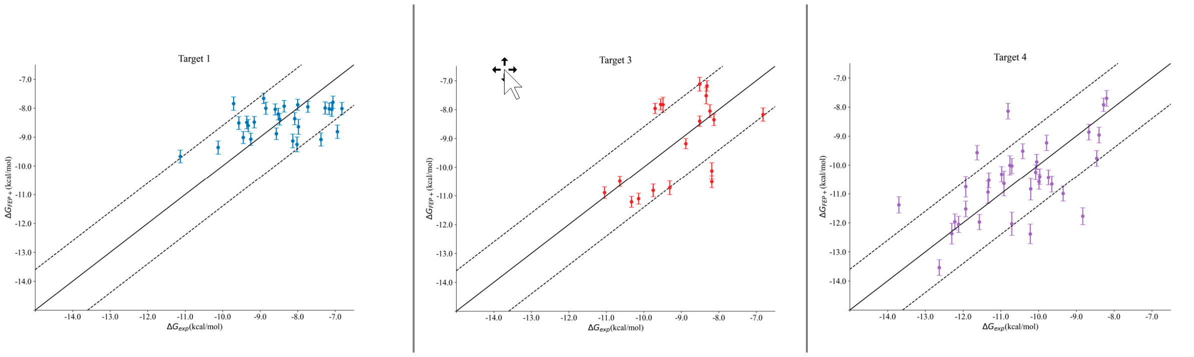

Figure S2. Correlation plots obtained from FEP+ for Targets 1, 3 and 4.

Table S3. Details of each run for DeltaDeltaG for each target

Dataset	Train	Test	R (run1)	R (run2)	R (run3)	R (run4)	R (run5)	R (average)	R (stdev)
Target 1	52	29	0.53	0.59	0.40	0.51	0.51	0.51	0.07
Target 2-Dataset1	72	51	0.86	0.86	0.85	0.86	0.87	0.86	0.01
Target 2-Dataset2	158	38	0.85	0.86	0.88	0.87	0.87	0.87	0.01
Target 3	57	20	0.35	0.61	0.55	0.55	0.59	0.53	0.11
Target 4	195	34	0.48	0.54	0.45	0.44	0.48	0.48	0.04
